# The Influence of the Amphiphilic Properties of Peptides on the Phosphatidylinositol Monolayer in the Presence of Ascorbic Acid

**DOI:** 10.3390/ijms252312484

**Published:** 2024-11-21

**Authors:** Iwona Golonka, Izabela W. Łukasiewicz, Aleksandra Sebastiańczyk, Katarzyna E. Greber, Wiesław Sawicki, Witold Musiał

**Affiliations:** 1 Department of Physical Chemistry and Biophysics, Faculty of Pharmacy, Wroclaw Medical University, Borowska 211A, 50-556 Wrocław, Poland; iwona.golonka@umw.edu.pl (I.G.); izabela.w.lukasiewicz@student.umw.edu.pl (I.W.Ł.); aleksandra.sebastianczyk@student.umw.wroc.pl (A.S.); 2 Department of Physical Chemistry, Faculty of Pharmacy, Medical University of Gdańsk, Al. Gen. J. Hallera 107, 80-416 Gdańsk, Poland; katarzyna.greber@gumed.edu.pl (K.E.G.); wieslaw.sawicki@gumed.edu.pl (W.S.)

**Keywords:** Langmuir isotherm, compression reversibility factor, antibacterial peptides, ascorbic acid, phosphatidylinositol membrane

## Abstract

*Acne vulgaris* is one of the most common dermatological diseases and is strongly connected with the pathological growth of the *Cutibacterium acnes*. More than half of the cultures of this bacterium are resistant to antibiotics, resulting in the proposal of the use of antibacterial peptides as an alternative to traditional antibiotics. Ascorbic acid (AA) and its antioxidant properties may ally in acne therapy. The aim of this study was to determine the influence of the selected antibacterial peptides in the presence of ascorbic acid and 3-O-ethyl-ascorbic acid (EAA) on the properties of the monolayer formed by phosphatidylinositol. Studies of the properties of the phosphatidylinositol monolayer were carried out using the Langmuir–Wilhelmy balance. The recorded compression isotherms, hysteresis loops, and surface pressure values recorded at specific time intervals were evaluated to assess the influence of ascorbic acid and its derivatives in the presence of antimicrobial peptides on the stability and organization of phosphatidylinositol monolayers. The addition of AA to the subphase caused a faster phase transition at over 60 Å^2^/molecule and significantly reduced the plateau surface pressure by about 20% in most of the systems tested. The studied monolayers were found to be in the expanded liquid state (40.23–49.95 [mN/m]) or in the transition between the expanded and condensed liquid phase (51.47–60.98 [mN/m]). Compression and decompression isotherms indicated the highest flexibility of the systems at 20 °C and 25 °C. The surface pressure versus time dependence indicated the stability of the phosphatidylinositol monolayer with 3-O-ethyl–ascorbic acid and antimicrobial peptides up to 35 °C.

## 1. Introduction

The search for new drugs for skin infections is an ongoing and evolving process, and there is a need to improve existing therapies or evaluate new therapeutic countermeasures. The desired direction includes antimicrobial activity and reduced cytotoxicity, and, ideally, no cytotoxic effect in new topical peptides. From the studies we have published so far, we know that the peptides (WKWK)_2_-KWKWK-NH_2_ (P2 in our previous paper) and (C12)_2_-KKKK-NH_2_ (P4 in our previous paper) exhibit antioxidant properties and antimicrobial activity against *Staphylococcus aureus* [1]. Peptide P2 showed greater antioxidant properties than P4, which exhibited amphiphilic properties. The lack of aromatic amino acid residues may contribute to the reduced antioxidant activity of P4, which was confirmed in the case of peptides with large side groups, such as histidine with an imidazole group or tryptophan with an indole group [2,3,4,5]. Peptide 4, containing, among others, four lysine residues in the form of polylysine (KKKK), showed the highest antistaphylococcal activity among the other tested peptides. The MBEC value was recorded at 250 mg/L for P4, but a strong reduction in staphylococcal cell numbers was also observed after exposure to 62.5 mg/L of this peptide. Polylysine-containing peptides are known to affect membrane protein kinases, phosphatidylinositol kinases, and adenylate cyclase [6]. The sorption of P2 and P4 on polymer–bacterial cellulose (BC) produced by *Komagateibacter xylinu* confirmed the possibility of the local application of these peptides on the BC carrier. P2, with its distinct positive charge, showed remarkable antimicrobial activity against *S. aureus* and *C. acnes*. The above-mentioned compounds did not show cytotoxic activity against the fibroblast line [1]. So far, only the P4 peptide has been shown to have antifungal activity against *Candida albicans*, *Candida tropicalis*, and *Aspergillus niger*, and also has antibacterial activity against Gram-positive *Staphylococcus epidermidis*, *Bacillus subtilis*, and *Enterococcus faecalis*, and Gram-negative *Escherichia coli*, *Klebsiella pneumonia*, and *Pseudomonas aeruginosa* [7]. One of our hypotheses was that these peptides disrupt the integrity of the bacterial membrane.

Using Molecular Dynamics Simulation, it was demonstrated that peptides tend to form a monolayer at the water–vacuum interface. In the case of P4, this can be explained by the presence of aliphatic chains, which are hydrophobic in nature and oriented away from the water layer according to the calculations. The P2 system was characterized by the presence of the interconnected amino acids lysine and tryptophan, which are not separated from each other. “In silico”, this peptide penetrated the membrane much deeper and the coefficient of diffusion was 0.1067 × 10^−5^ [cm^2^/s], and for P4, this was 0.0878 × 10^−5^ [cm^2^/s]. The surface tension in the peptide systems was as follows: 1116.22 [bar × nm] and 934.92 [bar × nm]. The optimal ability to penetrate the living layers of the epidermis is possessed by substances of medium lipophilicity with a maximum logP value of 1.0–3.0 [8]. In our case, P2 meets these requirements. To facilitate the administration of drugs through the skin, penetration enhancers are routinely used, which would be appropriate for the use of surfactant P4 [9].

Therefore, the Langmuir method was chosen as the main research method, which allows the observation of the behavior of peptides in the aqueous subphase in relation to a monolayer composed of phosphatidylinositol as a model cell membrane. Phosphatidylinositol, as a component of lipid membranes, plays significant roles in cell function. Depending on the inositol ring substitution, these forms can affect endocytosis, exocytosis, signal transduction, or ion channel function [10]. Phosphatidylinositol, in the presence of triacylglycerol and lipids, is a component of the cell envelope of *Cutibacterium acnes.* The cell wall of this bacterium has peptidoglycan, which, due to the presence of D-alanine and L-diaminopelic acid in the peptide chain, significantly distinguishes it from other G+ bacteria [11,12]. Ascorbic acid performs many important functions in the functioning of the body by influencing its biochemical and biological functions [13,14]. Therefore, ascorbic acid (AA) and 3-O-ethyl-ascorbic acid (EAA) occur in the studied systems in the aqueous subphase together with the peptides.

Previous studies have shown how the sequence of amino acids constituting the peptide, which also determines its spatial structure, influences its physicochemical properties and, thus, further therapeutic possibilities, which change depending on the environment.

The aim of this study was to determine the effect of peptide amphiphilicity in the presence of AA and EAA on the properties of a monolayer formed of phosphatidylinositol using the Langmuir method. According to the positive charge of P2 and P4 molecules, the assessed peptides may attract the anionic phosphatidylinositol groups of the model membrane with different strengths. The simultaneous application of the peptides with AA or its derivative EAA to the subphase resulted in the decreased surface pressure of the monolayer. The parallel result was the enlargement of the surface area assigned to one molecule of PI. This unexpected result of the shift of the surface per molecule to higher values was in contradiction to the possible facilitation of the incorporation of peptides into the monolayer, which requires further study.

## 2. Results

### 2.1. Compression Isotherms of the Monolayer Formed from PI with Peptides, AA, and EAA in the Aqueous Subphase

The compression isotherms of the phosphatidylinositol monolayer in the presence of P2 or P4 and AA or EAA in the aqueous subphase are presented in Supplementary Figures S1–S4. The measurements were performed at 20 °C, 25 °C, 30 °C, and 35 °C. For the phosphatidylinositol monolayer in the aqueous subphase, the pressure increase above zero at all four temperatures was observed at a surface area per molecule of about 103.70 Å^2^/molecule. The pressure at which the collapse was recorded for the PI monolayer at 20 °C was 38.82 mN/m at a surface area of 12.37 Å^2^/molecule; at 25 °C—39.17 mN/m at 17.72 Å^2^/molecule; at 30 °C—35.15 mN/m at 22.67 Å^2^/molecule; and at 35 °C—30.06 mN/m at 12.52 Å^2^/molecule. By comparing the isotherms of PI monolayers with EAA and AA in the aqueous subphase, it can be seen that the PI monolayers with EAA at temperatures of 20 °C and 30 °C reached collapse at higher values of surface pressure compared to the PI monolayers with AA. At 35 °C for the PI monolayer with AA, the surface pressure was higher at the moment of collapse than for the PI with EAA. The collapse values for PI–P2 and PI–P4 at 30 °C and 35 °C were the highest compared to the other systems. The increase in surface pressure above zero for the phosphatidylinositol monolayer with P4 and EAA and AA at four temperatures occurred at an average surface area of 103.66 Å^2^/molecule. Adding AA and EAA to the subphase in these systems caused a shift of the compression isometric towards higher surfaces as well as a decrease in the surface collapse pressure compared to the situation in which only P2 and P4 occurred in the subphase. At each temperature, the PI monolayers with P4 and EAA reached collapse at higher surface pressure values and a lower surface area per molecule compared to the PI monolayers with P4 and AA.

### 2.2. Compressibility Coefficient of the Monolayer Formed from PI with Peptides, AA, and EAA in the Aqueous Subphase

The dependence of the compressibility coefficient of the phosphatidylinositol monolayer as a function of the surface area per molecule of AA or EAA and P2 or P4 in the aqueous subphase at 20 °C, 25 °C, 30 °C, and 35 °C is shown in Figures S5–S8. For systems with EAA and then with AA, the compressibility coefficient achieved the highest values at a higher area per molecule compared to the other systems. The maximum values of the compressibility coefficient are given in Table 1. In the PI+P2+AA and PI+P4+AA systems, the compressibility coefficient exceeded 50 mN/m at all temperatures, which indicates that they were in a condensed liquid phase. The remaining systems with values of this parameter from 12.5 mN/m to 50 mN/m were in the expanded liquid state. In the case of PI+P2+EAA and PI+P4+EAA, an increase in the compressibility coefficient was observed compared to the systems without EAA.

**Figure 1:**
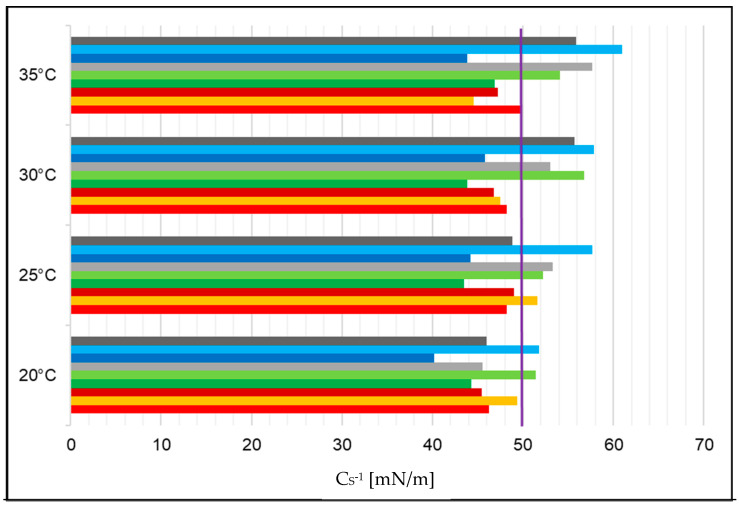
Compressibility coefficient C_S_^−1^ [mN/m] values of the tested systems: PI (−), PI+AA (−), PI+EAA (−), PI+P2 (−), PI+P2+AA (−), PI+P2+EAA (−), PI+P4 (−), PI+P4+AA (−), and PI+P4+EAA (−) at temperatures of 20 °C, 25 °C, 30 °C, and 35 °C. The purple line indicates the boundary between the expanded liquid phase and the condensed liquid phase.

In Figure 1, the purple line separates the systems that were in the expanded liquid state from the condensed liquid phase. It can be seen that the least stable systems were observed at 20 °C. With increasing temperature, the PI, PI+P2, PI+P2+EAA, PI+P4+AA, and PI+P4+EAA systems stabilized.

### 2.3. Compression and Expansion of the Monolayer Formed from PI with Peptides, AA, and EAA in the Aqueous Subphase

The compression and decompression isotherms for the PI monolayer in the water subphase with AA and EAA at temperatures of 20 °C, 25 °C, 30 °C, and 35 °C are presented as three hysteresis loops, as shown in Figures S9–S12. At temperatures of 20 °C, 25 °C, and 30 °C, the hysteresis of the PI monolayer in the water subphase with AA and EAA exhibited a similar shape, and the width of each successive loop decreased to a small extent. The situation was similar at 35 °C, with the hysteresis of the PI monolayer with AA clearly shifting towards smaller surfaces. At all temperatures, decompression did not follow the compression path and the range in which the hysteresis occurred was within the surface pressure range from about 0 to 33.4 mN/m. The course of the hysteresis of the monolayer formed from PI with peptides, AA, and EAA in the aqueous subphase is shown in Figures S13–S16. In the course of the hysteresis at 30 °C and 35 °C, the decompression did not follow the compression. At both temperatures, a similar loop shape could be observed, which was characterized by an emerging flattening on the compression line. The hysteresis of the PI monolayer over P2 and EAA at 30 °C occurred in a larger range of surface pressure compared to the hysteresis of the PI monolayer over P2 and AA. The hysteresis of the PI monolayer in the PI+P4, PI+P4+AA, and PI+P4+EAA systems is shown in Figures S17–S20. At the first three temperatures, the hysteresis of the PI monolayer over P4 shifted toward smaller areas compared to the hysteresis for the PI+P4+AA and PI+P4+EAA systems. At 35 °C, the compression and decompression for the three hysteresis loops were close to each other for the PI+P4 system. The successive hysteresis loops of the PI+P4+EAA system ran close to each other at all temperatures.

### 2.4. Dependence of Surface Pressure on Time of the Monolayer Formed from PI with Peptides, AA, and EAA in the Aqueous Subphase

**Figure 2:**
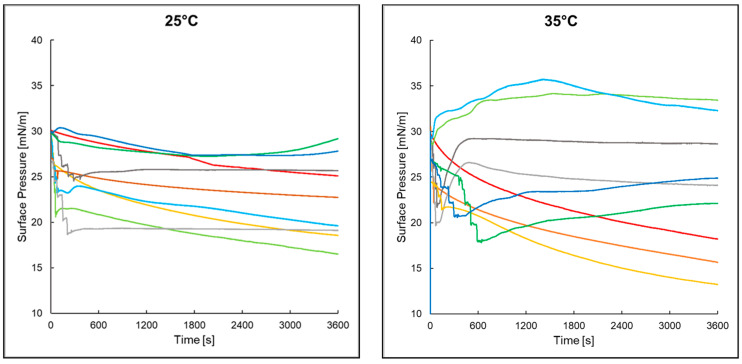
Dependence of surface pressure on time at temperatures of 25°C and 35°C of the tested systems: PI (—), PI+AA (—), PI+EAA (—), PI+P2 (—), PI+P2+AA (—), PI+P2+EAA (—), PI+P4 (—), PI+P4+AA (—), and PI+P4+EAA (—).

For the PI monolayer in the water subphase at both 25 °C and 35 °C, a continuous decrease in surface pressure was observed over time (Figure 2). For the PI monolayer over P2 in the water subphase at 25 °C, a decrease in surface pressure over 35 min to a value of about 27.20 mN/m was observed, and then it increased to a value of 29.20 mN/m for the remaining 25 min of measurement. At 35 °C, an increase in the surface pressure was observed over 20 min to a value of about 34.00 mN/m, and then it maintained this value at an approximately constant level for the next 40 min. For the phosphatidylinositol monolayer over peptide 4 in the water subphase at 25 °C, an increase in the surface pressure to a value of 30.40 mN/m was observed in the 2nd minute of measurement, and then it decreased to a value of 27.4 mN/m in the 28th minute of measurement. Then, the pressure was maintained at a constant level until the 60th minute of measurement. The difference between these pressures was 3 mN/m. At 35 °C, however, an increase in the surface pressure was observed for 25 min to a value of 35.70 mN/m, and then it decreased to a value of 32.30 mN/m for the next 35 min. The addition of AA to the water subphase at 25 °C caused a large decrease in surface pressure in the systems both at the beginning of the measurement and over time. The situation was different in the case of adding EAA to the water subphase, where the changes in the surface pressure over time were smaller or even stabilized. The addition of P2 and P4 to the aqueous subphase caused the surface pressure of the monolayer to initially decrease and then increase, which was slower with time. The addition of AA and EAA to the water subphase at 35 °C caused a large increase in the surface pressure in the systems with P2 and P4 and its stabilization over time.

## 3. Discussion

**Figure 3:**
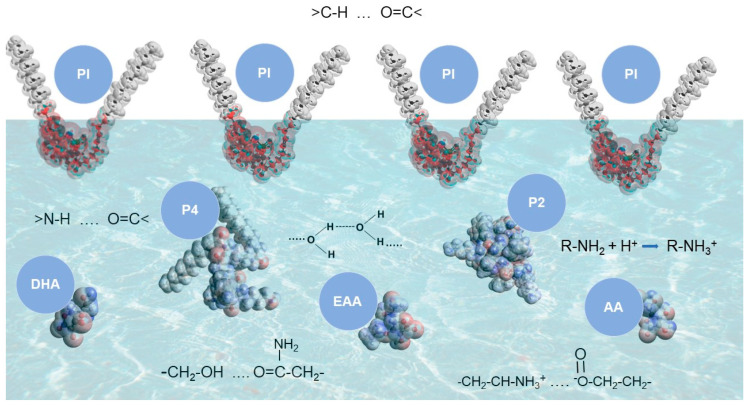
Scheme of phase boundary in Langmuir balance, on which a monolayer of phosphatidylinositol (PI) was formed in an aqueous subphase. P2, P4, AA, or EAA were introduced into the subphase in the systems assessed in the experiments—the acronyms and the evaluated systems are presented in Table 2. Dehydroascorbic acid (DHA) is an oxidized form of ascorbic acid. The molecules were imaged using an electrostatic surface. From this surface, it is possible to interpret where the electron density is the highest—red areas—and where the electron density is the lowest—dark blue areas [20].

Due to the increasing resistance of bacteria, including *C. acnes*, to the antibiotics used so far, new methods for treating bacterial infections are being sought [15]. As the newly synthesized peptides (WKWK)_2_-KWKWK-NH_2_ and (C12)_2_-KKKK-NH_2_ are known to have many physicochemical properties that can potentially be used to create prospective drugs, their effect on a monolayer composed of a component of *Cutibacterium* membranes, i.e., phosphatidylinositol, was investigated. By analyzing the course of the surface pressure dependence on the surface area per molecule in the form of π-A isotherms, it was possible to determine the interactions between the phosphatidylinositol molecules in the monolayer and the components of the aqueous subphase. Initially, compression isotherms were measured for the phosphatidylinositol monolayer, which served as a reference for the remaining monolayers with the addition of AA and EAA and antimicrobial peptides. The addition of only the P2 and P4 peptides to the subphase shifted the compression isotherm toward smaller surfaces, which may be the result of interactions with the groups of the monolayer that favor higher organization (Figure 3) [16]. The lack of a decrease in the surface pressure at which the plateau begins, observed in the PI–P4 system, may be explained by the fact that P4 has the most lipophilic character compared to the other peptides. This high lipophilicity is determined by two hydrophobic chains in its structure, which, in the case of a highly fluidified monolayer, may penetrate between the phospholipid molecules in an attempt to “get out” to the water surface [17]. In the expanded liquid phase, the PI monolayer provides enough space so that P4’s hydrophobic interactions with the monolayer can occur more easily. Consequently, in the case of the condensed liquid phase, the main effect is peptide binding and the subsequent condensation of the monolayer [18]. The interactions between the peptide and phospholipid molecules lead to the significant reorientation or even disintegration of the ordered structure of the peptide and play a condensing role, leading to increased ordering of the alkyl chain in the phospholipid monolayer [19].

The PI monolayers with EAA in the subphase at all temperatures were characterized in most systems by isotherms similar to the isotherms of the PI monolayers in the water-only subphase. However, for the PI monolayers with AA in the subphase at 25 °C and 30 °C, a decrease in surface pressure was observed. This decrease in the surface pressure of the compression isotherm at which the plateau begins and the initiation of this phenomenon on a larger surface area per molecule, e.g., for the PI+AA, PI+P2, PI+P2+AA, and PI+P4+AA systems in comparison to the phospholipid monolayer alone, may indicate a faster initiation of the phase transition from the expanded liquid phase to the condensed liquid phase [21]. The shift of the isotherms towards larger surfaces after the addition of peptides to the subphase and for systems mainly involving AA may indicate that the peptides were incorporated into the phosphatidylinositol monolayer [22]. This phenomenon may be explained by the formation of hydrogen bonds in the system via the hydroxyl groups of the ascorbic acid [23]. The NH_2_ group can be protonated in the case of ascorbic acid and interact with the polar PI head. Ascorbic acid has two acidic protons on the C2- and C3-enol hydroxyl groups (pKaC3 = 4, pKaC2 = 11.19) conjugated to its C1-carbonyl group [24]. In acidic solutions, ascorbic acid can be deprotonated to form ascorbate. Ascorbate is an excellent reducing agent and readily undergoes two successive single-electron oxidations to form the ascorbate radical and then dehydroascorbic acid (DHA, Figure 3). At pH 3, i.e., below the pKa of ascorbic acid, the protonated form predominates, but a small fraction can be deprotonated to form DHA. In the case of EAAs, one hydroxyl group (pKaC2 = 8.19) is protected by a substituent; therefore, the degradation mechanism leading to DHA should not be possible [24]. In this way, the substituent stabilizes the molecules, which will be less reactive to degradation reactions. The measurements were performed at four temperatures, where, for example, heating the water in the subphase increases the solubility of AA in water [25]. EAA and peptides are stable in this temperature range. However, weaker bonds can form between the phosphatidylinositol molecules in the monolayer, allowing for the easier penetration of P4 and P2 into the monolayer. The increase in the number of specific molecules in the monolayer may favor the increase in the observed surface pressure of the monolayer.

In order to determine the physical state of the tested monolayers, the compressibility coefficient calculated from the dependence of the surface pressure on the surface area per molecule was analyzed. From the obtained results, it can be concluded that most of the monolayers occur in the expanded liquid phase, as their maximum compressibility coefficient values are in the range of 12.5–50.0 mN/m [26,27]. The remaining monolayers are in a transition state between the expanded liquid phase and the condensed liquid phase, as evidenced by their maximum compressibility coefficient values in the range of 50–100 mN/m [28]. Comparing this parameter between the phosphatidylinositol monolayer (water) and the phosphatidylinositol monolayers with EAA and AA, it can be seen that it does not change significantly. A different situation occurs for the PI monolayers with EAA and AA—in the presence of antimicrobial peptides, the value of this parameter increases, which may indicate the stabilization of the monolayer [29]. For the PI system with EAA and P4, and the PI system with AA and P4, the compressibility coefficient increases with increasing temperature, which indicates an increase in the degree of its stiffness and, thus, a decrease in its elasticity [30].

In all of the systems studied, the shift of each successive hysteresis loop towards lower surfaces is noticeable, which may be the result of the monolayer particles being more tightly packed [31]. Alternatively, some of the particles could pass into the water subphase. The smallest loop shifts and distances between the compression and decompression are characteristic of monolayers at temperatures of 20 °C and 25 °C. The small loops were mainly observed in the monolayers structured over the P4 peptide solutions, indicating the high stability of the monolayer [32].

In order to determine the stability of the phosphatidylinositol monolayer with ascorbic acid and its derivatives in the presence of antimicrobial peptides, a surface pressure–time dependence measurement was performed. For the PI monolayers with EAA and P4 at 25 °C and for PI with AA and P4 at 35 °C, a continuous increase in pressure was observed, which may indicate the penetration of peptides into the phosphatidylinositol monolayer [33]. In the case of the remaining monolayers, a continuous slow decrease in surface pressure was visible at 25 °C, indicating a possible desorption of phosphatidylinositol molecules into the aqueous subphase [34]. For PI monolayers with EAA and peptides and for PI monolayers with AA and P2 at 35 °C, an increase in the surface pressure was observed for about 6 min, after which the pressure stabilized at a certain level, which suggests that the systems stabilized during the measurement.

The introduction of peptides (WKWK)_2_-KWKWK-NH_2_ and (C12)_2_-KKKK-NH_2_ and of AA and EAA into the subphase affects the organization of the PI molecules of the monolayer at the liquid/air interface. This causes a change in the properties of its surface depending on the structure of the peptide, the structure of ascorbic acid, and the temperature at which the experiment was conducted. The physical state of the monolayer is influenced by interactions between these molecules in the surface layer and molecules in the subphase. Their strength and range change as a result of the approach or separation of molecules. In our studies, it was observed that the addition of peptides increased the surface pressure of the phosphatidylinositol monolayer, similarly to previous studies of the lecithin monolayer [17]. The addition of AA to the subphase decreased the surface pressure of the monolayer of systems with peptides, which could be expected. The same was true for the addition of EAA, but the effect was much weaker. However, the shift of the isotherms of these systems towards larger surfaces was surprising, and may have facilitated the incorporation of peptides into the monolayer. The surface pressure parameters, compressibility, and reversibility of the monolayer compression can be used to rationally design antimicrobial agents with selective toxicity to specific organisms.

Antimicrobial peptides (AMPs) are short peptides of 10 to 50 amino acids. Although their lengths, sequences, and conformations vary, they share some common features. Typical AMPs consist of positively charged residues such as arginine, lysine, or histidine. A cationic peptide with a net positive charge ranging from +2 to +11 can interact with microbial membranes [35,36,37,38]. Peptide P2 and P4 meet these requirements. In addition to the undeniable advantages of AMP, there are also limitations related to susceptibility to proteolytic degradation, binding to serum proteins, decrease in antibacterial activity in the presence of physiological salt concentration, and production costs resulting from the complexity of the structure [39]. The possible transdermal transport of the peptides may potentially result in internal immunogenic activity. Thus, tests considering inflammatory reactions may also be included in future evaluations of preparations based on peptides.

## 4. Materials and Methods

### 4.1. Synthesis and Characterization of Peptides

**Figure 4:**
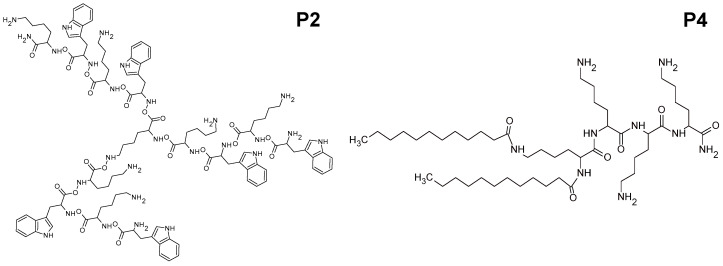
The structure of peptides P2 (WKWK)_2_-KWKWK-NH_2_ and P4 (C12)_2_-KKKK-NH_2_.

The preparation, purification, and determination of the structure of the peptides (Figure 4) were carried out as described in our earlier publication [9].

### 4.2. Langmuir Films

To study monolayers formed from phosphatidylinositol (PI) on an aqueous subphase with P2 and P4 in the presence of AA or EAA, a Langmuir–Wilhelmy trough manufactured by Kibron Microtrough X. in Helsinki (Finland), together with the accompanying Filmware X 4.0 computer software, was used. The balance consists of a tetrafluoroethylene (Teflon) tray measuring 23.7 cm long and 7.9 cm wide, two movable Teflon barriers, and a wire (used instead of a Wilhelmy plate) weighing 48.2 mg and 0.5 mm in diameter made of platinum, which ensures a negligible contact angle during the measurement. In order to avoid the introduction of impurities, before each measurement, the platinum plate was rinsed with methanol and then with water, and ignited in a burner flame. The barriers moved at a speed of 10 mm/min. The cleanliness of the surface of the carrier phase was checked by measuring the surface tension during the movement of the railings towards the center of the tank. If the value of the voltage change did not exceed 0.30 mN/m, the surface was considered free of impurities. Otherwise, the washing procedure was repeated. The tubs were placed on anti-vibration tables. All measurements were performed in three repetitions. A Kruss thermostat (Hamburg, Germany) was used to maintain a constant temperature of 20 °C, 25 °C, 30 °C, and 35 °C during the measurements. The abovementioned temperature values were selected according to the literature data [40] and reflect the range of temperatures that may be observed on different surfaces of the human body in the vicinity of the facial region, as well as in the ambient environment of the air surrounding the human body.

### 4.3. Compression Isotherms of Phosphatidylinositol on the Aqueous Subphase with P2 or P4 and AA or EAA

After checking the purity of the subphase, chloroform (Merck, Darmstadt, Germany) and methanol (Merck, Darmstadt, Germany) were mixed in a ratio of 9:1, and then phosphatidylinositol (C_47_H_82_NaO_13_P, Sigma-Aldrich, St. Louis, MO, USA) was dissolved to obtain a solution of 15 μL in volume and a concentration of 1.18 × 10^−6^ mol/L, which was applied to the surface of the water subphase. After the evaporation of the solvent, the monolayer was symmetrically compressed to a surface pressure of 5 mN/m, and then 30 μL of a 1.27 × 10^−3^ mol/L aqueous solution of the tested peptide was introduced into the subphase, as well as 30 μL of AA or EAA solution of 1.27 × 10^−3^ mol/L. The composition of the studied systems and respective abbreviations are given in Table 2. After 15 min of the system stabilizing, the barriers were slid off inwards at a speed of 10.02 mm/min. The force acting on the wire is expressed using the following formula [41]:

$$F={⍴}_{g}g{\pi r}^{2}l+2\gamma \left(\pi r\right)cos\theta -{⍴}_{l}{\pi r}^{2}h$$

where F—net force [N], ⍴_g_—wire density [kg/m^3^], ⍴_l_—density of the subphase [kg/m^3^], g—gravitational constant [n/kg], r—wire radius [m], l—wire length [m], h—insertion depth of the wire [m], γ—the surface tension of the liquid [mN/m], and θ—contact angle.

### 4.4. Hysteresis

The monolayers were compressed and decompressed to obtain hysteresis plots, which were realized analogously to the above simple recordings of compression isotherms. The same speed was used for moving and spreading the barrier (10 mm/min). However, in this case, the three loops were recorded in every measurement. In the Filmware X 4.0 program, the measurement range was determined on the basis of isotherm compression.

### 4.5. Compressibility Coefficient of the Monolayer

Compressibility, C_S_, is a parameter that can be calculated from the course of π-A isotherms and is commonly used to characterize monolayers. However, the concept of the compressibility modulus, compressibility factor, or compressibility coefficient is more commonly used and is presented as reciprocal of compressibility [42]:

$$C_{S}^{-1}=-A\left(\frac{d\pi }{dA}\right)$$

where C_S_^−1^—compressibility factor [mN/m], A—surface area per molecule [Å^2^/molecule], and π—surface pressure [mN/m].

The use of the compressibility coefficient C_S_^−1^ in describing the elasticity of Langmuir monolayers is often practiced due to the convenient range of its value in this type of experiment, since the compressibility values (C_S_) are fractions of the magnitude 10^−2^–10^−3^ [42].

### 4.6. Surface Pressure Changes over Time

In this experiment, 15 μL of phosphatidylinositol solution was placed in the aqueous subphase and left for 15 min until the solvent had evaporated. Subsequently, the monolayer was compressed to the pressure range of natural biological membranes, i.e., of 30 mN/m. Then, 30 μL of the assessed antibacterial peptide and 30 μL of AA or EAA were added to the aqueous subphase. The surface pressure was measured at selected temperatures, 25 °C and 35 °C, through the entire period of 60 min immediately after the completion of the subphase with AA or EAA, as a function of time over a constant area.

## 5. Conclusions

Due to the cationic nature of P2 and P4, these peptides may be attracted by anionic phosphatidylinositol groups of the model membrane at different strengths. Depending on the amphiphilicity of the peptide, the temperature of the system, and the presence of AA or EAA in the subphase, the molecular organization and packing of molecules in the monolayer can change in different ways. Considering the compressibility coefficient results, the most ordered monolayer was that of the system that included peptides and AA. With the increase in temperature, in most cases, the compressibility coefficient increased. The research results can be used for the rational design of antimicrobial agents acting against *Cutibacterium acne*. The tests should be supplemented with microbiological tests of systems and anti-inflammatory effects.

## Figures and Tables

**Table 1:** Compressibility coefficient C_S_^−1^ [mN/m] values of the tested systems.

Acronym	Temperature			
	20 °C	25 °C	30 °C	35 °C
PI	46.23	48.18	48.22	49.95
PI+AA	49.39	51.58	47.53	44.60
PI+EAA	45.47	49.01	46.77	47.21
PI+P2	44.28	43.50	43.81	46.86
PI+P2+AA	51.47	52.26	56.78	54.12
PI+P2+EAA	45.58	53.32	53.33	57.71
PI+P4	40.23	44.19	45.80	43.84
PI+P4+AA	51.79	57.71	57.85	60.98
PI+P4+EAA	45.97	48.85	55.68	55.89

**Table 2:** The composition of the systems studied by the Langmuir–Wilhelmy method.

Acronym	Composition [Molecules]				
	PI (Monolayer)	AA (Subphase)	EAA (Subphase)	P2 (Subphase)	P4 (Subphase)
PI	1.20 × 10^16^				
PI+AA	1.20 × 10^16^	2.30 × 10^16^			
PI+EAA	1.20 × 10^16^		2.30 × 10^16^		
PI+P2	1.20 × 10^16^			2.30 × 10^16^	
PI+P2+AA	1.20 × 10^16^	2.30 × 10^16^		2.30 × 10^16^	
PI+P2+EAA	1.20 × 10^16^		2.30 × 10^16^	2.30 × 10^16^	
PI+P4	1.20 × 10^16^				2.30 × 10^16^
PI+P4+AA	1.20 × 10^16^	2.30 × 10^16^			2.30 × 10^16^
PI+P4+EAA	1.20 × 10^16^		2.30 × 10^16^		2.30 × 10^16^

## Data Availability

The scientific data are available at Wroclaw Medical University, Department of Physical Chemistry and Biophysics.

## Supplementary Materials

The following supporting information can be downloaded at: https://www.mdpi.com/article/10.3390/ijms252312484/s1.
