# Supplementary material for: The Influence of the Amphiphilic Properties of Peptides on the Phosphatidylinositol Monolayer in the Presence of Ascorbic Acid
Source: Int J Mol Sci. 2024 Nov 21;25(23):12484. doi: 10.3390/ijms252312484 (PMC11640797; doi:10.3390/ijms252312484)
Supplement: Supplementary file 1 [file ijms-25-12484-s001.zip › ijms-3291491-supplementary.pdf]

Supplementary

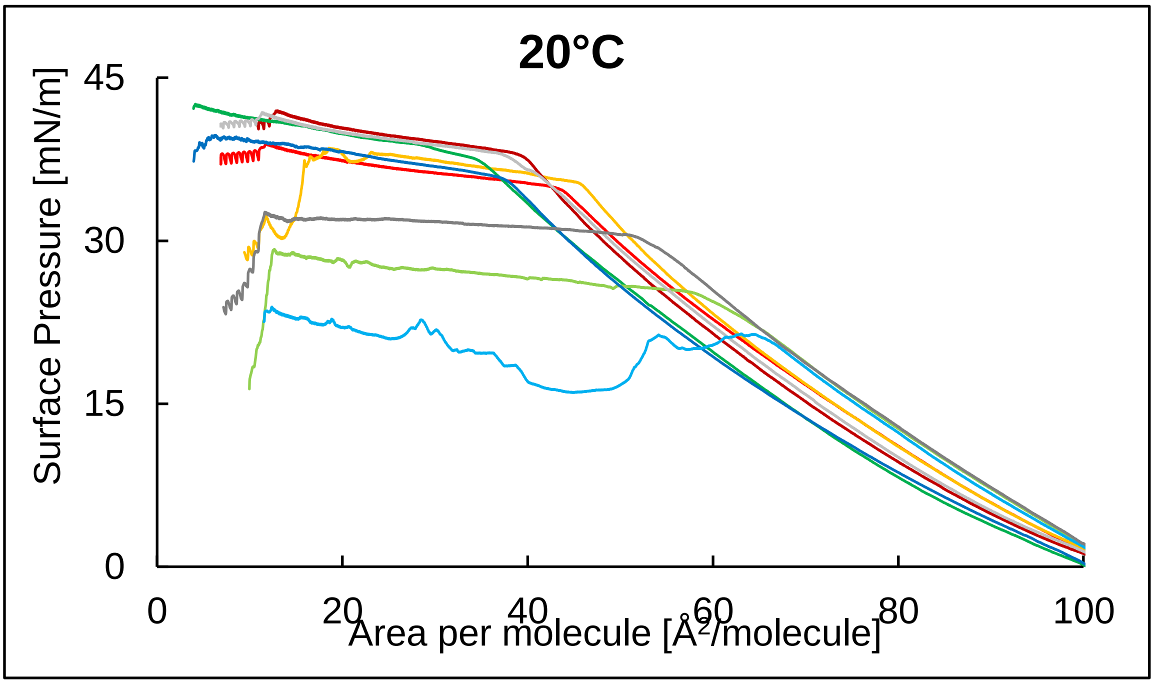

Figure S1. The course of the compression isotherms of the systems PI (—), PI+AA (—), PI+ EAA (—), PI+P2 (—), PI+P2+AA (—), PI+P2+ EAA (—), PI+P4 (—), PI+P4+AA (—), PI+P4+ EAA (—) at temperature of 20°C.

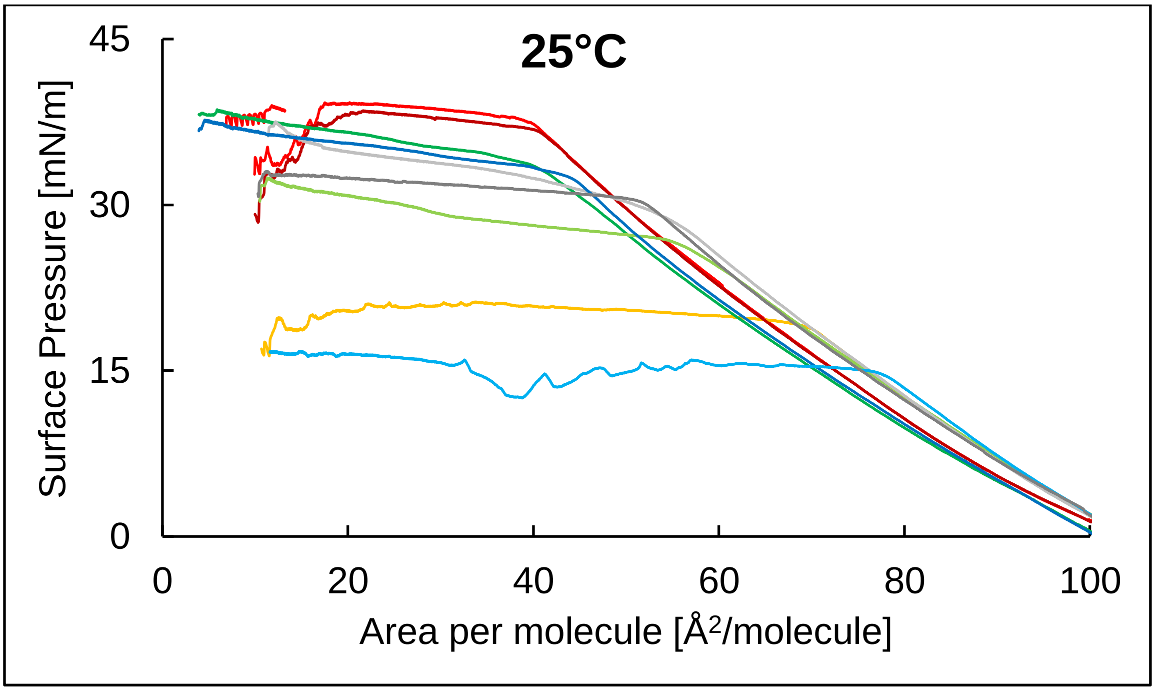

Figure S2. The course of the compression isotherms of the PI (—), PI+AA (—), PI+ EAA (—), PI+P2 (—), PI+P2+AA (—), PI+P2+ EAA (—), PI+P4 (—), PI+P4+AA (—), PI+P4+ EAA (—) at temperature of 25°C.

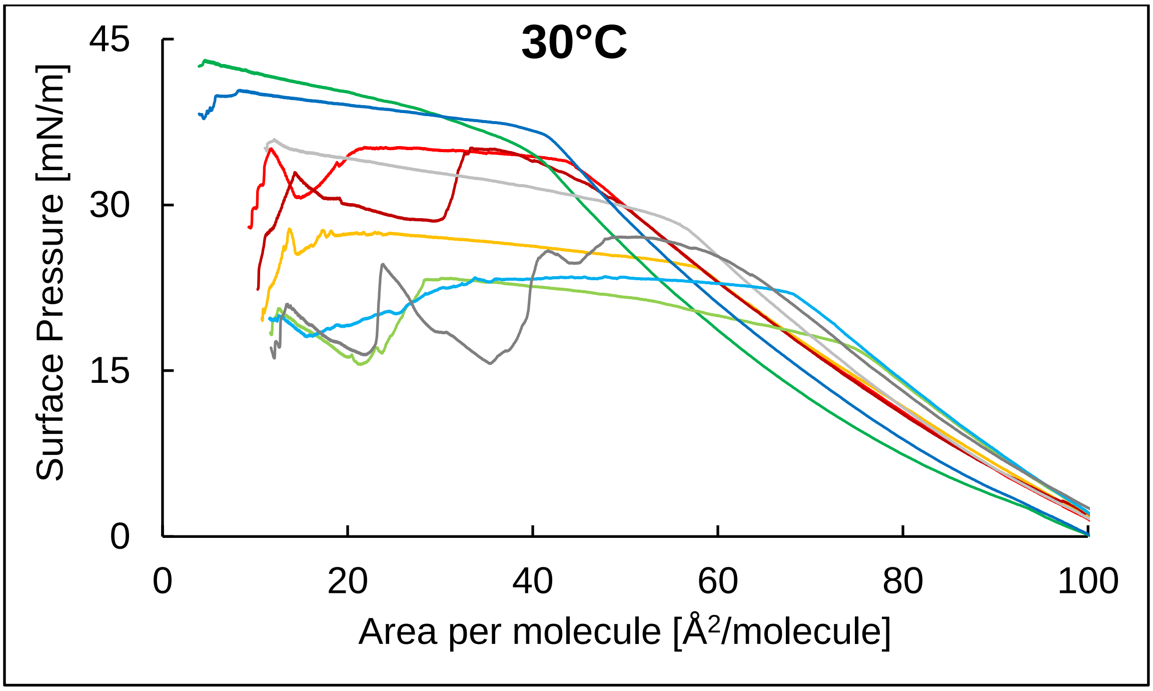

Figure S3. The course of the compression isotherms of the systems PI (—), PI+AA (—), PI+ EAA (—), PI+P2 (—), PI+P2+AA (—), PI+P2+ EAA (—), PI+P4 (—), PI+P4+AA (—), PI+P4+ EAA (—) at temperature of 30°C.

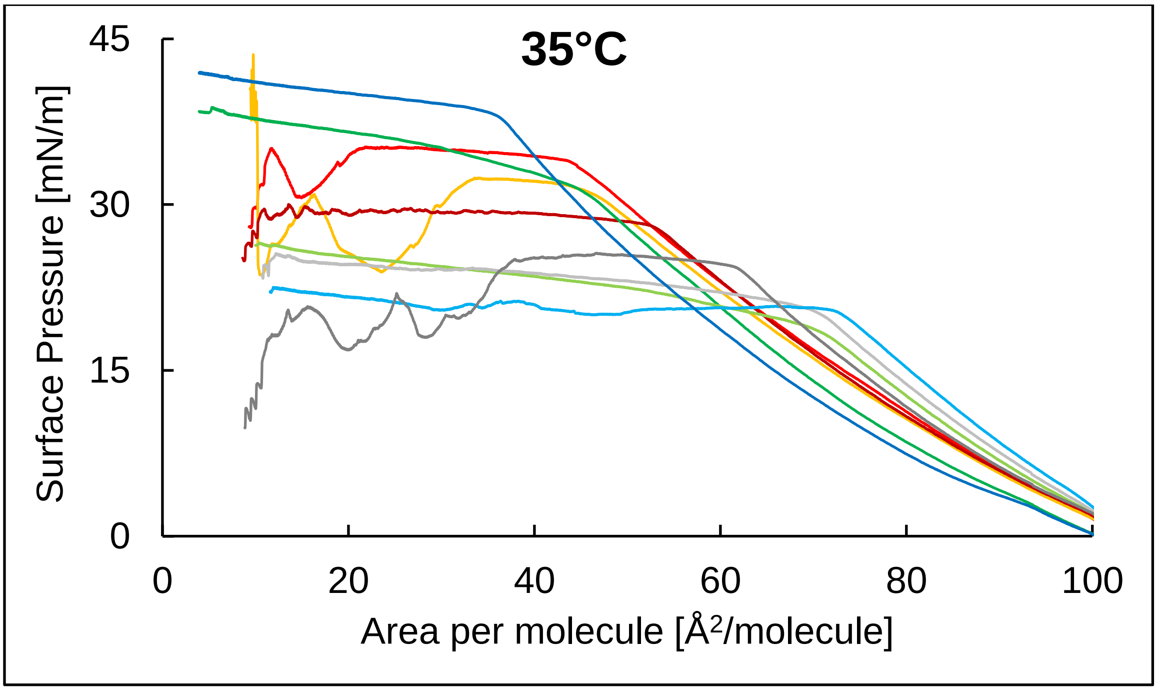

Figure S4. The course of the compression isotherms of the systems PI (—), PI+AA (—), PI+ EAA (—), PI+P2 (—), PI+P2+AA (—), PI+P2+ EAA (—), PI+P4 (—), PI+P4+AA (—), PI+P4+ EAA (—) at temperature of 35°C.

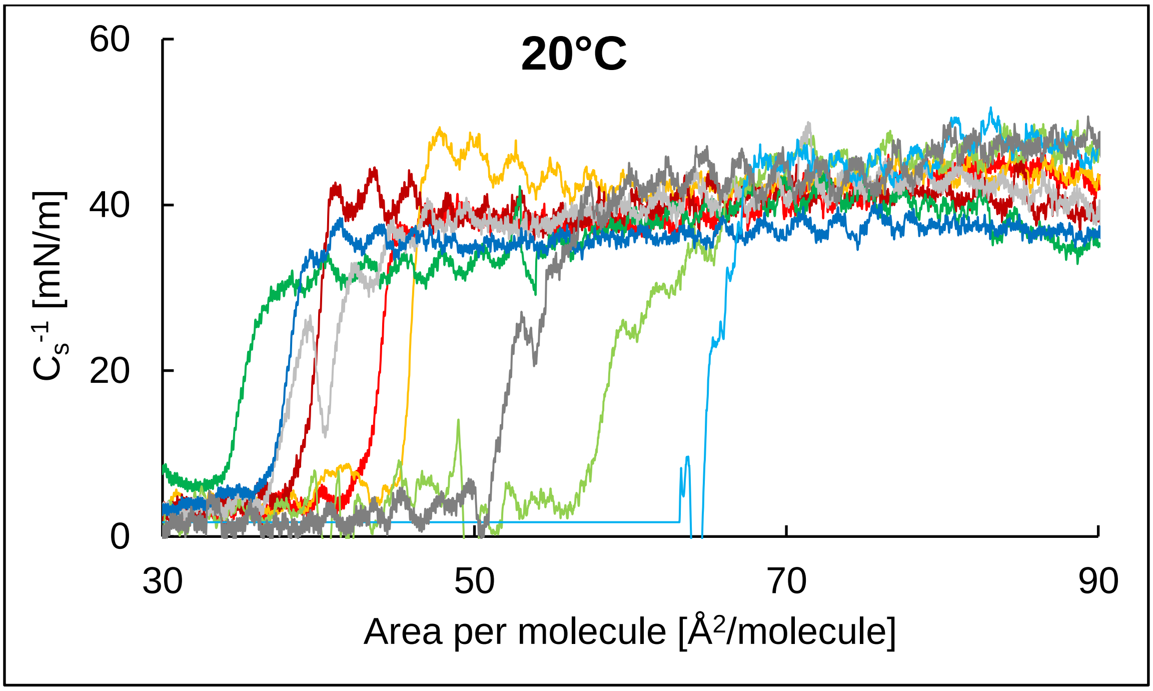

Figure S5. Dependence of the compressibility coefficient depending on the surface area per molecule of the systems PI (—), PI+AA (—), PI+ EAA (—), PI+P2 (—), PI+P2+AA (—), PI+P2+ EAA (—), PI+P4 (—), PI+P4+AA (—), PI+P4+ EAA (—) at temperature of 20°C.

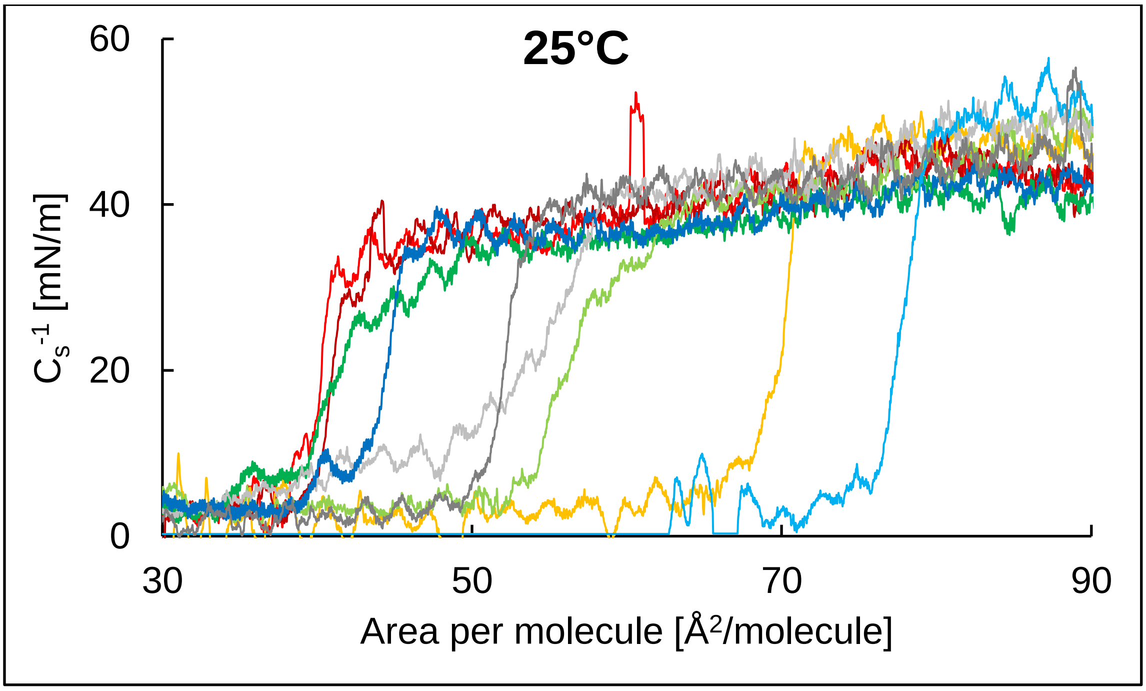

Figure S6. Dependence of the compressibility coefficient depending on the surface area per molecule of the systems PI (—), PI+AA (—), PI+ EAA (—), PI+P2 (—), PI+P2+AA (—), PI+P2+ EAA (—), PI+P4 (—), PI+P4+AA (—), PI+P4+ EAA (—) at temperature of 25°C.

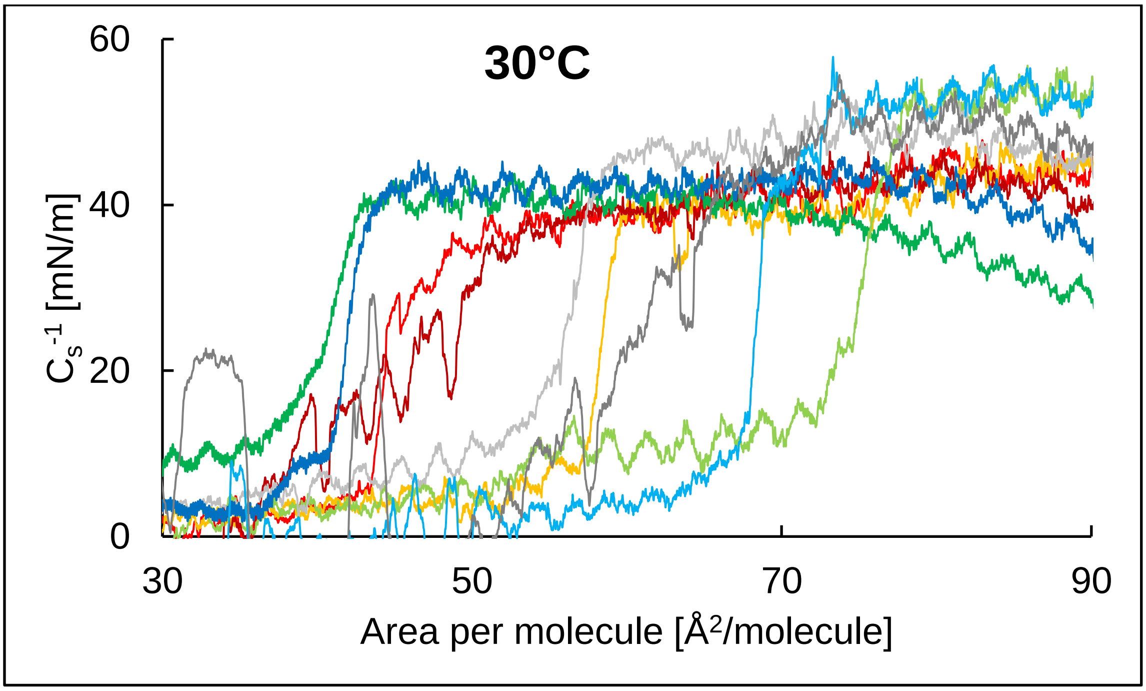

Figure S7. Dependence of the compressibility coefficient depending on the surface area per molecule of the systems PI (—), PI+AA (—), PI+ EAA (—), PI+P2 (—), PI+P2+AA (—), PI+P2+ EAA (—), PI+P4 (—), PI+P4+AA (—), PI+P4+ EAA (—) at temperatures of 30°C.

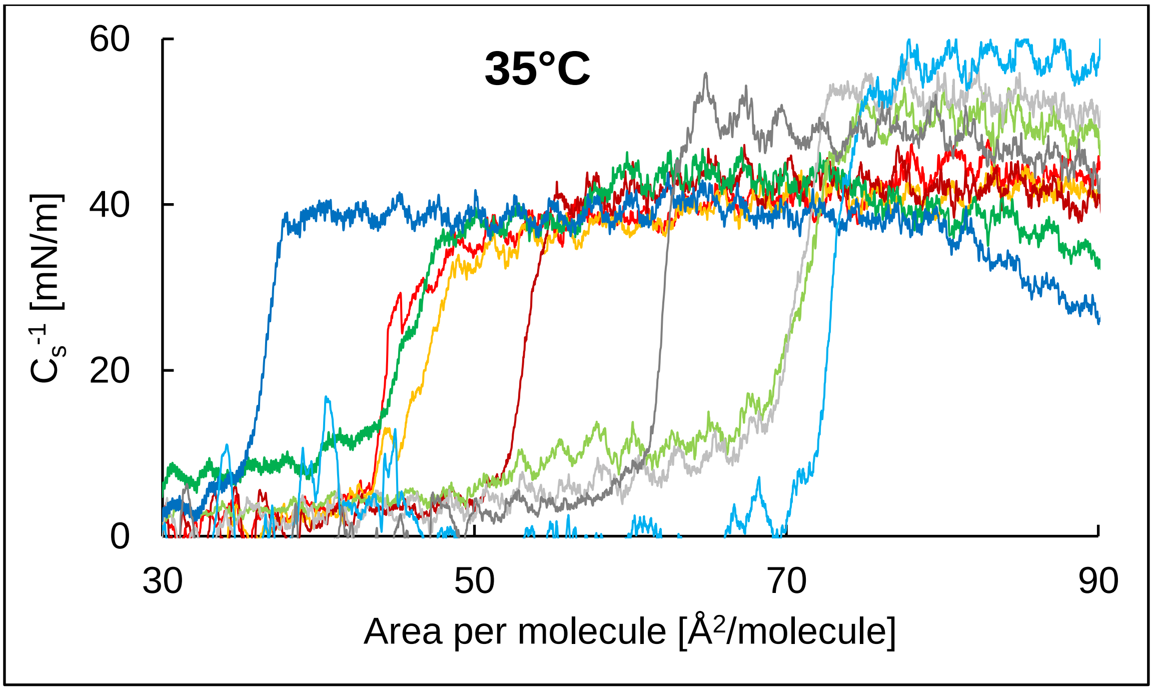

Figure S8. Dependence of the compressibility coefficient depending on the surface area per molecule of the systems PI (—), PI+AA (—), PI+ EAA (—), PI+P2 (—), PI+P2+AA (—), PI+P2+ EAA (—), PI+P4 (—), PI+P4+AA (—), PI+P4+ EAA (—) at temperature of 35°C.

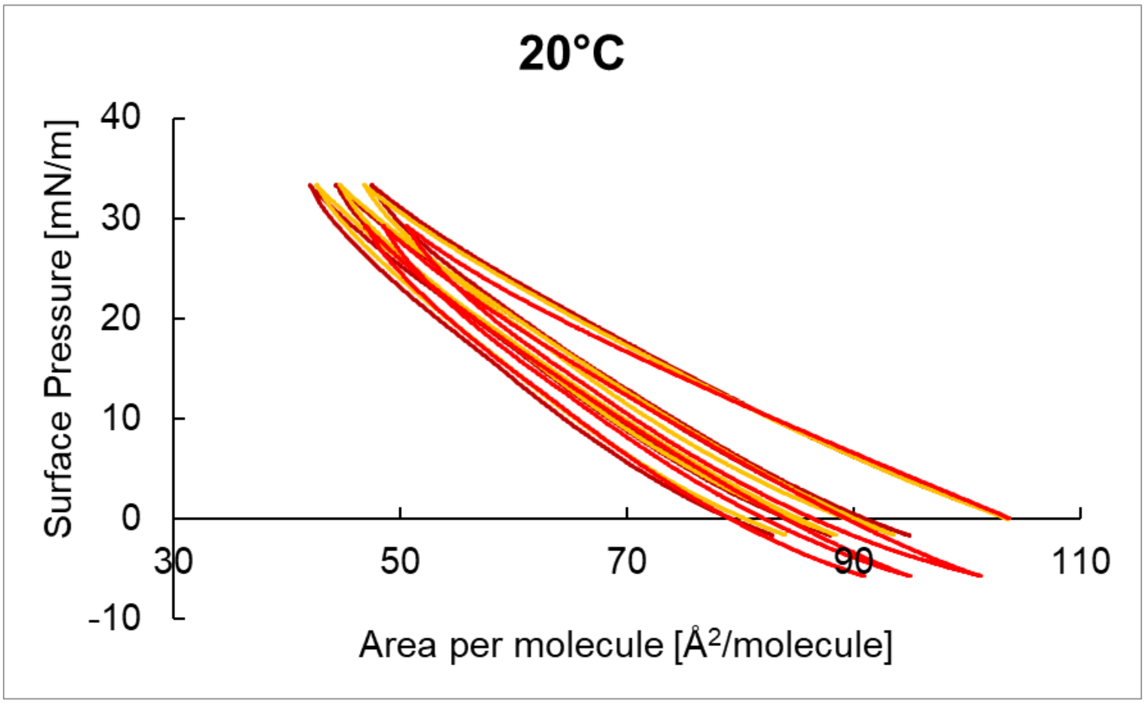

Figure S9. Hysteresis course of phosphatidylinositol PI monolayer (—) with ascorbic acid PI+AA (—) and 3-O-ethyl-ascorbic acid PI+EAA (—) in the aqueous subphase at temperatures of 20°C.

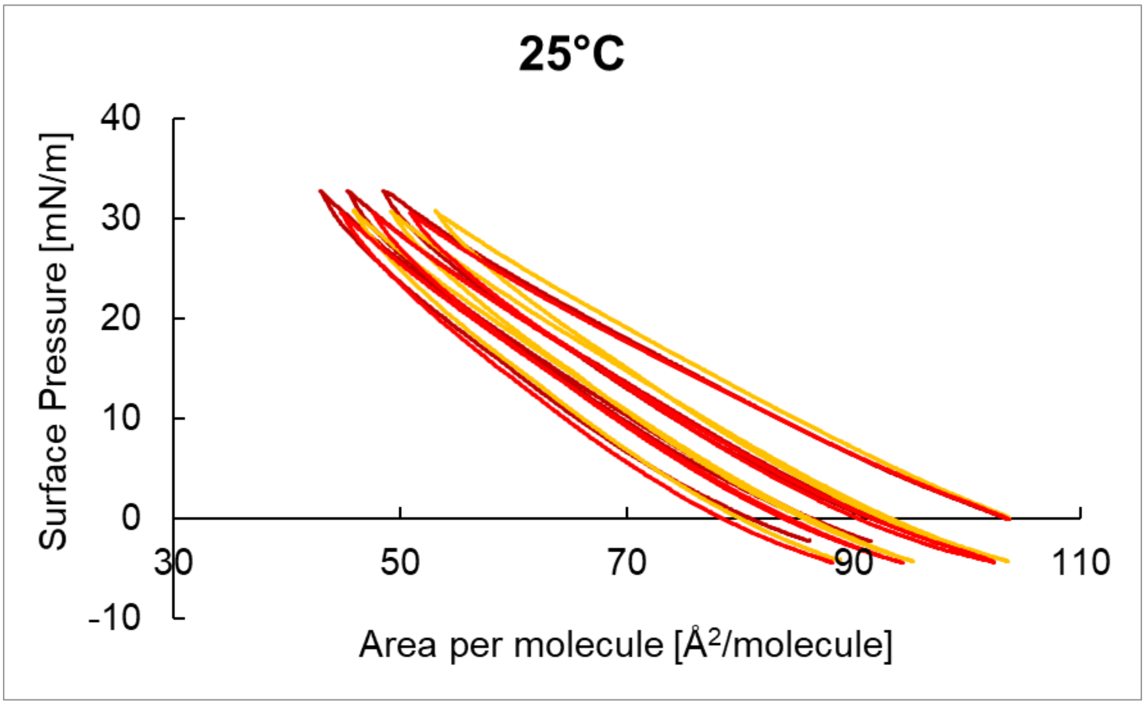

Figure S10. Hysteresis course of phosphatidylinositol PI monolayer (—) with ascorbic acid PI+AA (—) and 3-O-ethyl-ascorbic acid PI+EAA (—) in the aqueous subphase at temperatures of 25°C.

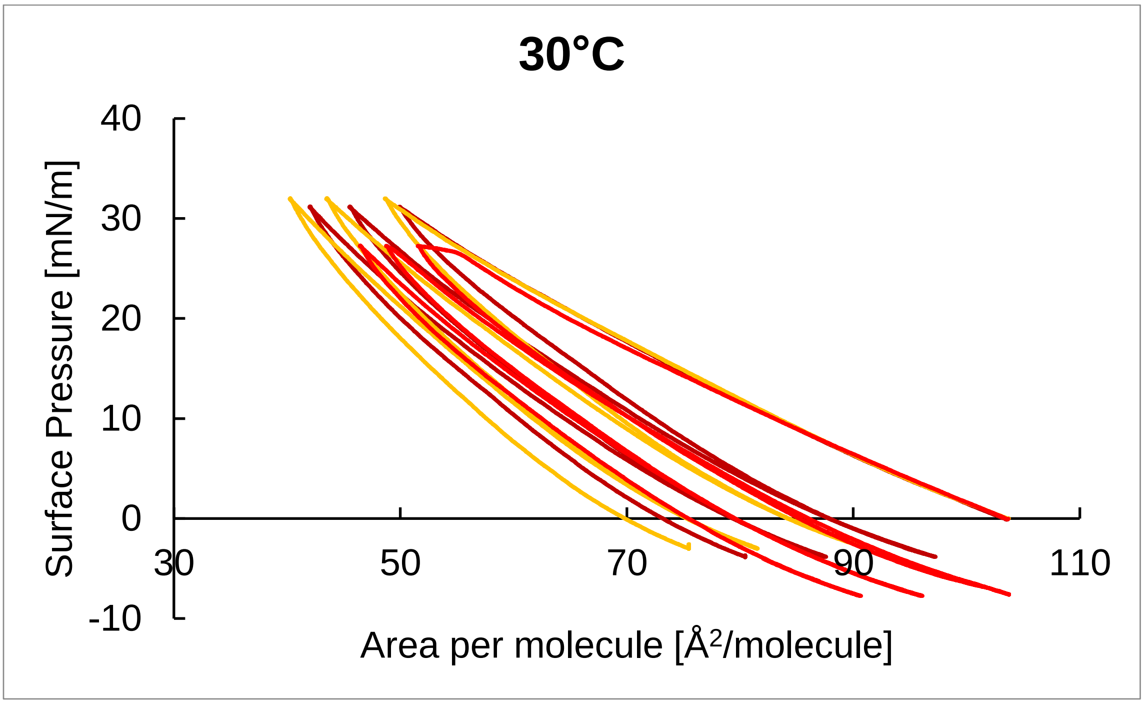

Figure S11. Hysteresis course of phosphatidylinositol PI monolayer (—) with ascorbic acid PI+AA (—) and 3-O-ethyl-ascorbic acid PI+EAA (—) in the aqueous subphase at temperatures of 30°C.

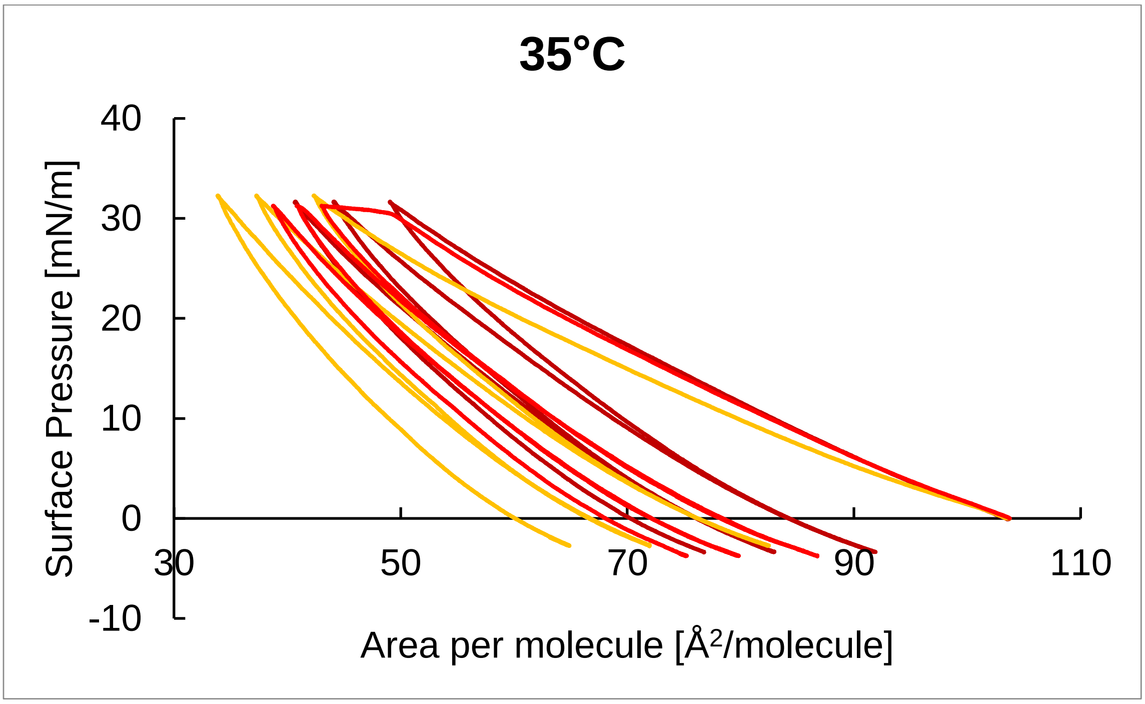

Figure S12. Hysteresis course of phosphatidylinositol PI monolayer (—) with ascorbic acid PI+AA (—) and 3-O-ethyl-ascorbic acid PI+EAA (—) in the aqueous subphase at temperatures of 35°C.

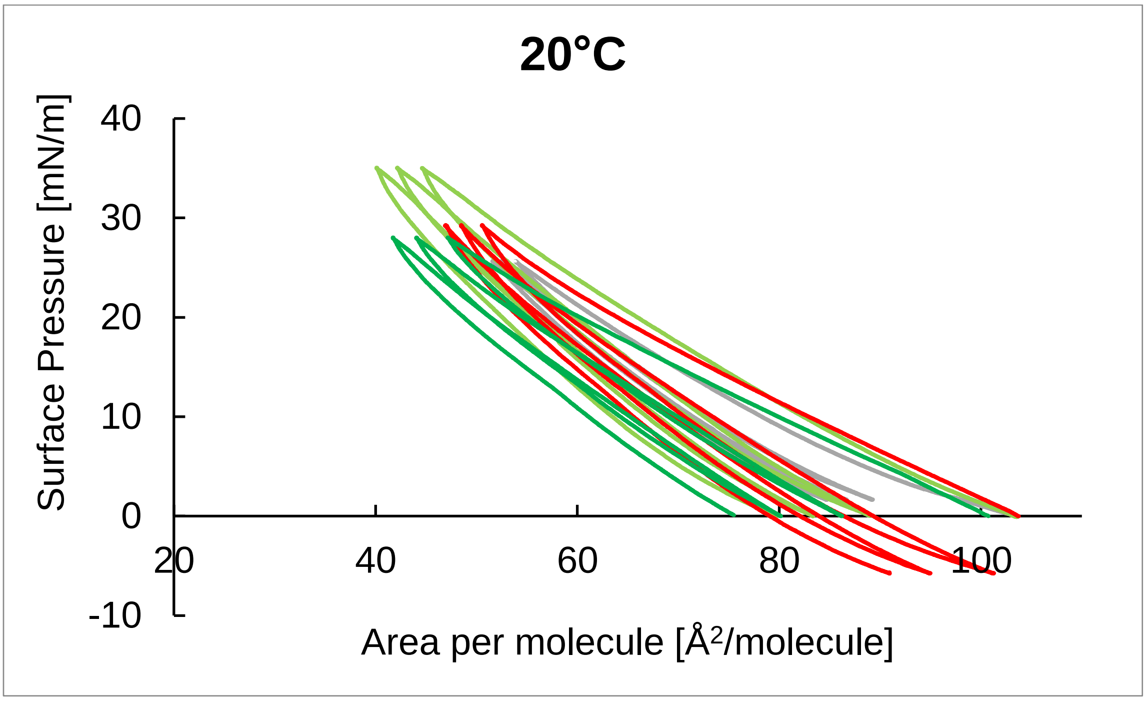

Figure S13. Hysteresis course of phosphatidylinositol PI monolayer (—) in the presence of P2; PI+P2 (—),ascorbic acid PI+P2+AA (—)and 3-O-ethyl-ascorbic acid PI+P2+EAA (—) in the aqueous subphase at temperatures of 20°C.

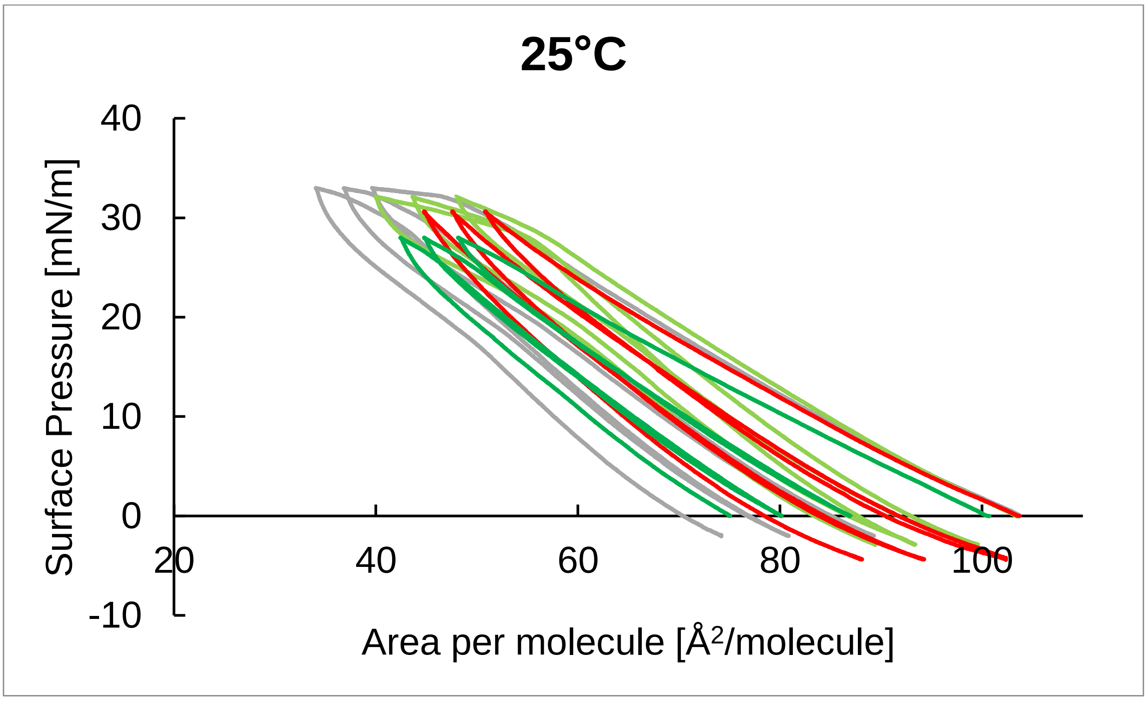

Figure S14. Hysteresis course of phosphatidylinositol PI monolayer (—) in the presence of P2; PI+P2 (—),ascorbic acid PI+P2+AA (—)and 3-O-ethyl-ascorbic acid PI+P2+EAA (—) in the aqueous subphase at temperatures of 25°C.

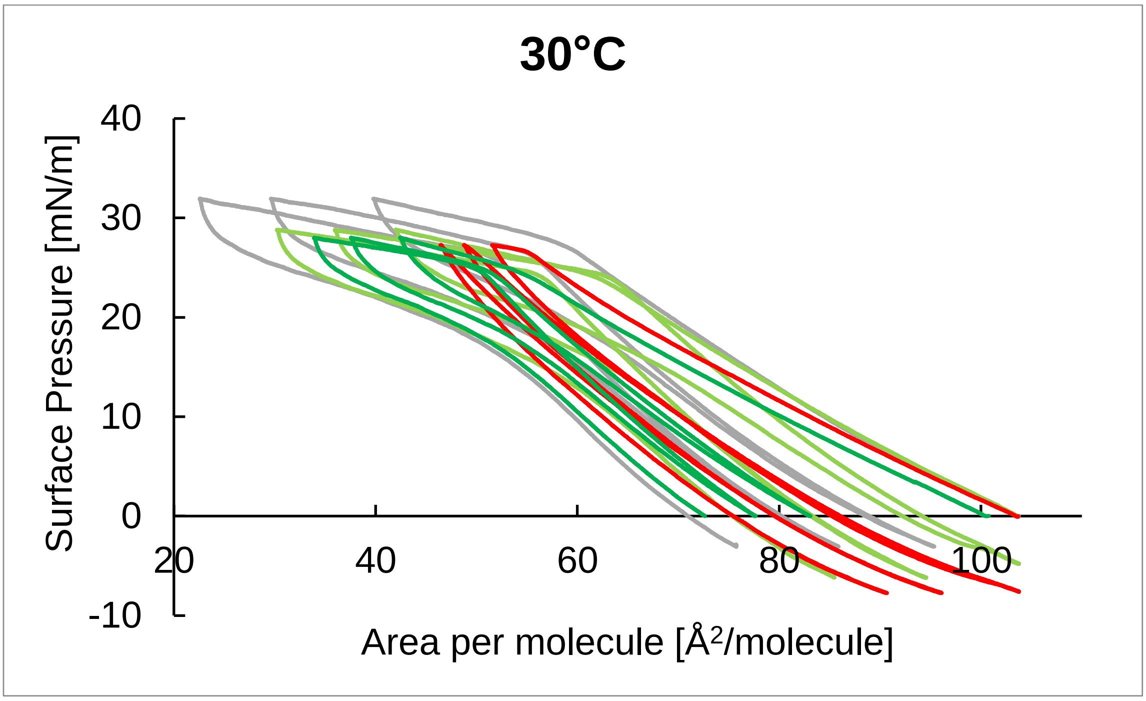

Figure S15. Hysteresis course of phosphatidylinositol PI monolayer (—) in the presence of P2; PI+P2 (—),ascorbic acid PI+P2+AA (—)and 3-O-ethyl-ascorbic acid PI+P2+EAA (—) in the aqueous subphase at temperatures of 30°C.

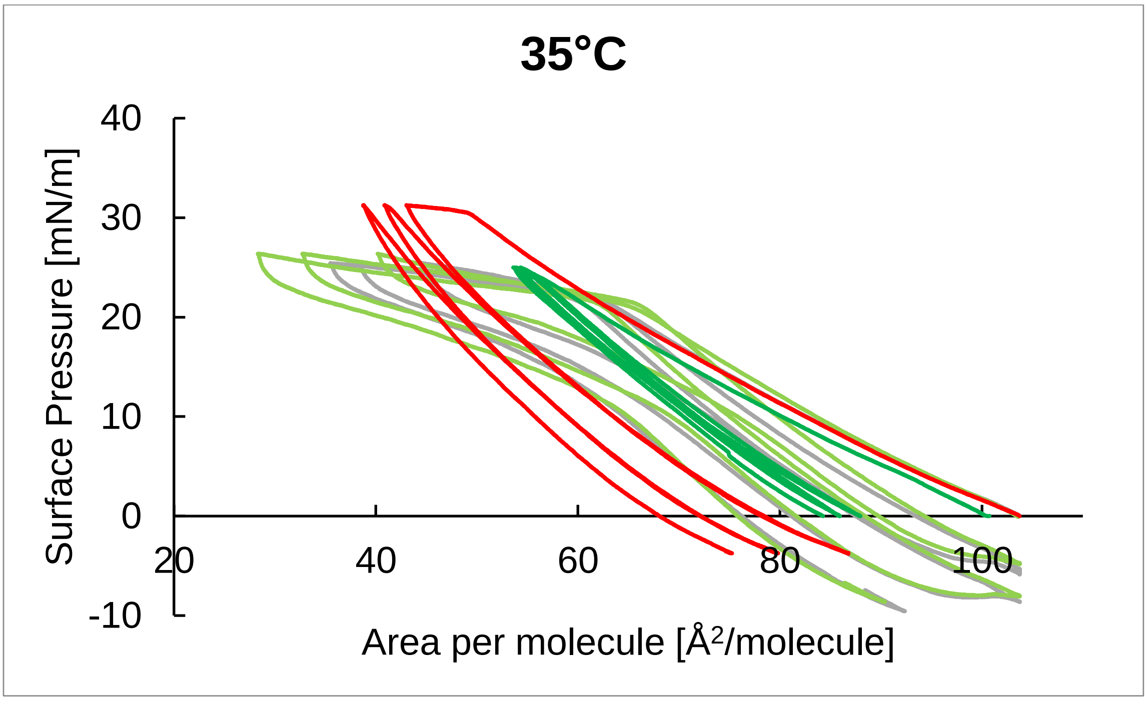

Figure S16. Hysteresis course of phosphatidylinositol PI monolayer (—) in the presence of P2; PI+P2 (—),ascorbic acid PI+P2+AA (—)and 3-O-ethyl-ascorbic acid PI+P2+EAA (—) in the aqueous subphase at temperatures of 35°C.

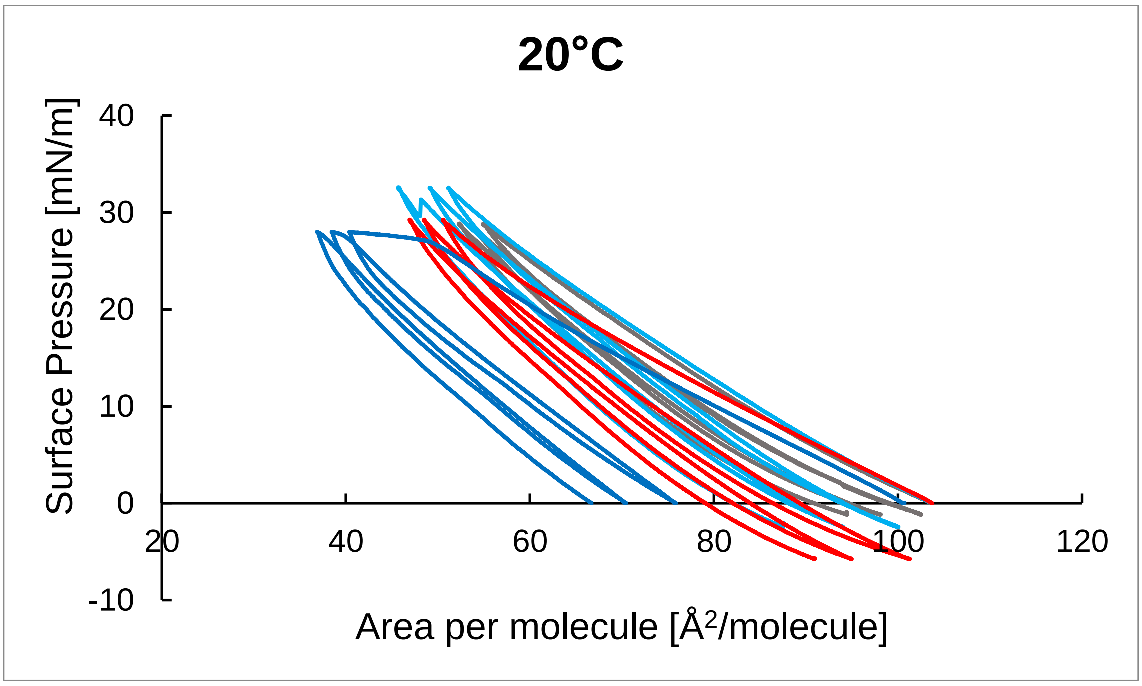

Figure S17. Hysteresis course of phosphatidylinositol PI monolayer (—) in the presence of P4; PI+P4 (—), ascorbic acid PI+P4+AA (—), and 3-O-ethyl-ascorbic acid PI+P4+ EAA (—) in the aqueous subphase at temperatures of 20°C.

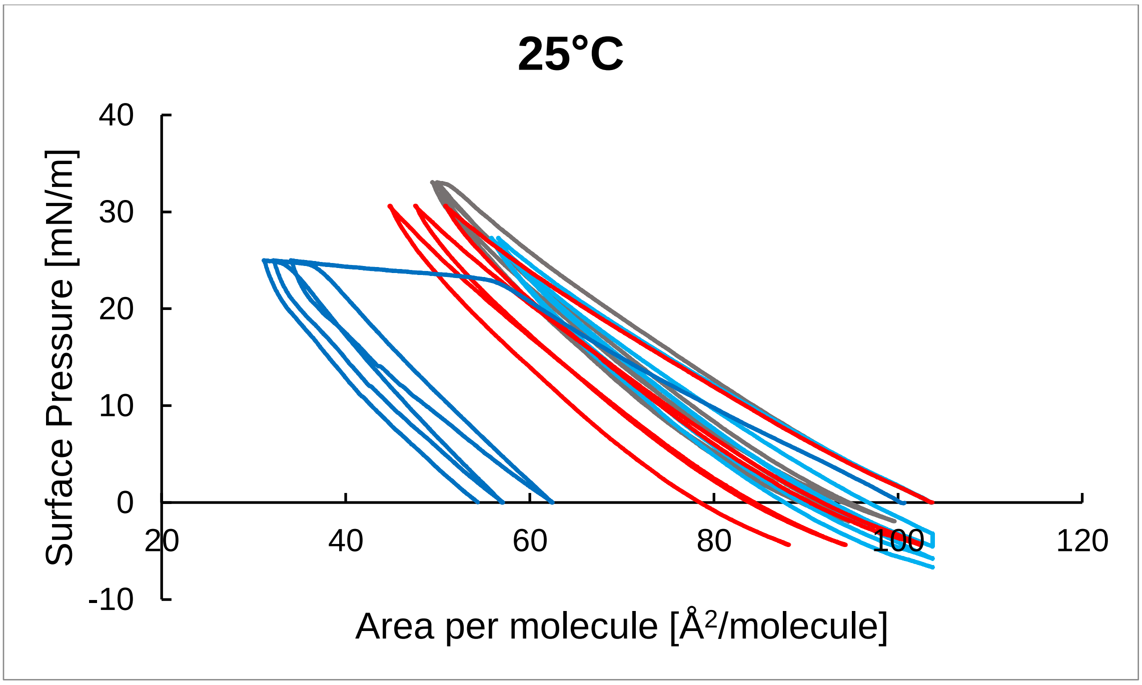

Figure S18. Hysteresis course of phosphatidylinositol PI monolayer (—) in the presence of P4; PI+P4 (—), ascorbic acid PI+P4+AA (—), and 3-O-ethyl-ascorbic acid PI+P4+ EAA (—) in the aqueous subphase at temperatures of 25°C.

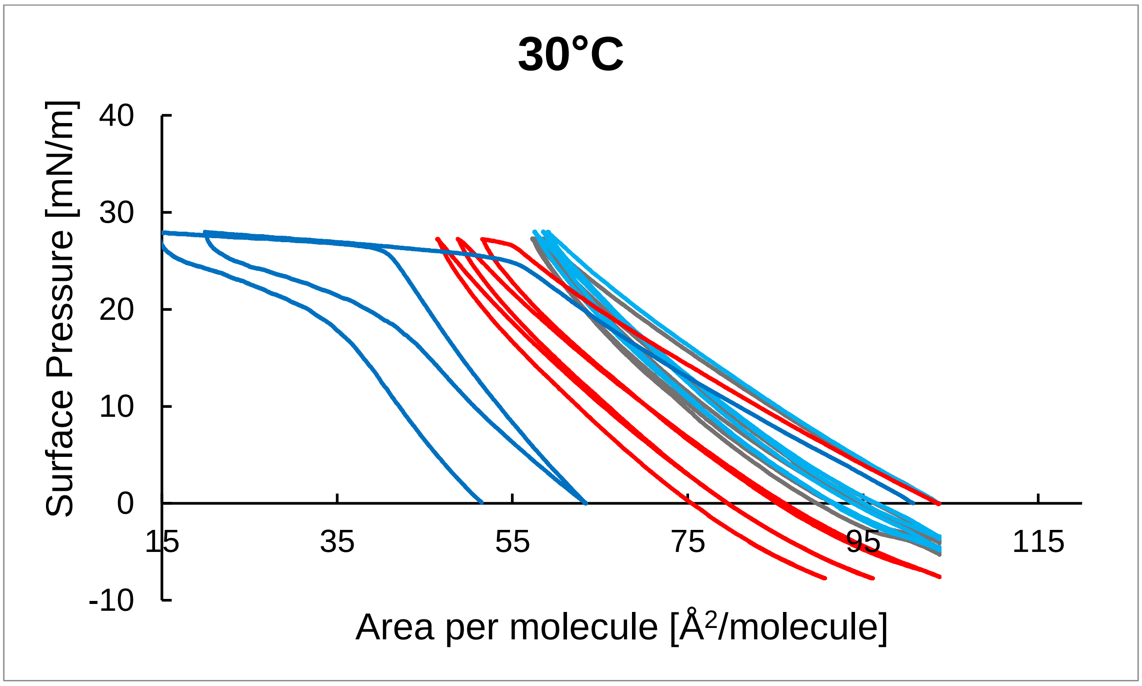

Figure S19. Hysteresis course of phosphatidylinositol PI monolayer (—) in the presence of P4; PI+P4 (—), ascorbic acid PI+P4+AA (—), and 3-O-ethyl-ascorbic acid PI+P4+ EAA (—) in the aqueous subphase at temperatures of 30°C.

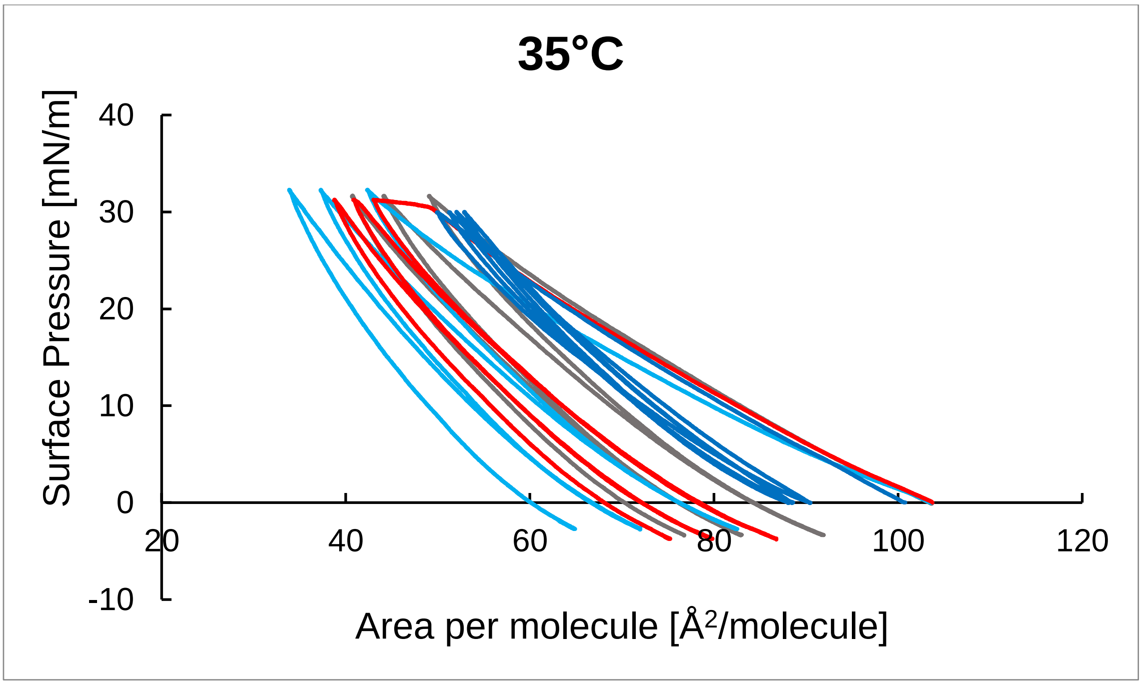

Figure S20. Hysteresis course of phosphatidylinositol PI monolayer (—) in the presence of P4; PI+P4 (—), ascorbic acid PI+P4+AA (—), and 3-O-ethyl-ascorbic acid PI+P4+ EAA (—) in the aqueous subphase at temperatures of 30°C.
